# Test‐Retest Reliability of Single Spectral Power and Spectral Power Ratios in Relative and Absolute Values

**DOI:** 10.1002/brb3.71035

**Published:** 2025-11-05

**Authors:** Jinwon Chang

**Affiliations:** ^1^ Department of Psychology Williams College Williamstown Massachusetts USA

**Keywords:** electroencephalography, resting state, spectral power, test–retest reliability

## Abstract

**Purpose:**

The current study provides the first systematic evaluation of the test‐retest reliability of electroencephalography (EEG) spectral power ratios alongside absolute and relative spectral power using two independent datasets. This approach aims to identify reproducible and clinically meaningful EEG features for longitudinal and diagnostic applications.

**Method:**

Two separate datasets of healthy adults were analyzed. Dataset 1 included 60 participants with EEGs recorded across three sessions (two on the same day and one month later), while Dataset 2 involved 74 participants recorded 1 h apart. Absolute, relative, and ratio‐based spectral features were computed across five brain regions using fast Fourier transform (FTP). Intraclass correlation coefficients (ICCs) were calculated to assess short‐ and long‐term test‐retest reliability.

**Finding:**

Spectral power ratios, particularly the alpha/beta ratio, consistently outperformed single‐band absolute and relative power features in reliability. Absolute alpha/beta ratios in the central, parietal, occipital, and temporal regions showed ICC values exceeding 0.75 across both datasets. These results were stable across different intervals and independent recording systems, demonstrating strong generalizability.

**Conclusion:**

This study demonstrates that spectral power ratios, especially the absolute alpha/beta ratio, are highly reliable EEG features. By leveraging two independent datasets, we establish their reproducibility across different populations and conditions. Our findings position spectral power ratios as robust, reproducible biomarkers for clinical EEG applications. This is the first study to rigorously validate their reliability across independent datasets, laying a foundation for their use in diagnostic and longitudinal monitoring tools.

## Graphical abstract

Channel‐wise intracorrelation coefficient of spectral power measures

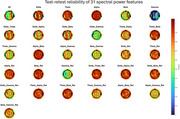

## 1 Introduction

Electroencephalography (EEG) is a widely utilized method for recording voltage fluctuations resulting from transmembrane ionic currents generated by synchronized neuronal populations. These fluctuations reflect diverse physiological processes, including postsynaptic potentials, dendritic resonance, calcium spikes, and intrinsic membrane currents (Buzsáki et al. 2012). EEG's high temporal resolution enables the detection of rapid neural dynamics, such as event‐related potentials (ERPs), which can be time‐locked to cognitive and behavioral events with millisecond precision. Consequently, EEG remains a powerful tool for studying brain–behavior relationships in both clinical and cognitive neuroscience.

Quantitative EEG (qEEG) extends traditional EEG by enabling statistical comparisons across individuals and groups. By shifting focus from expert‐dependent scoring to quantifiable neural markers, qEEG facilitates population‐level analysis and supports its integration in large‐scale cognitive and clinical investigations (Klimesch 1999). Among its analytic approaches, spectral power analysis and functional connectivity are two of the most widely employed.

Functional connectivity captures temporal correlations between signals from spatially distinct electrode sites, often using coherence or covariance measures. However, these indices exhibit limited test‐retest reliability and are highly sensitive to electrode configuration and frequency bands. In a study assessing test‐retest reliability across ten repeated EEG recordings, spectral power metrics showed substantially greater reliability than coherence, highlighting functional connectivity's vulnerability to spatiotemporal variability (Gudmundsson et al. 2007). Also, a review of multiple studies on EEG functional connectivity showed that measures of functional connectivity, including the phase lag index, demonstrated low test‐retest reliability across studies (Lopez et al. 2023).

In contrast, spectral power analysis offers a more stable and interpretable index of brain activity (Lopez et al. 2023; Tenke et al. 2018). Using fast Fourier transform (FFT), EEG signals are decomposed into sinusoidal components, enabling quantification of power within canonical frequency bands such as delta, theta, alpha, beta, and gamma. These band‐specific power values can then be statistically compared between conditions or groups, facilitating the identification of neural correlates of cognitive function or disease states.

Nevertheless, spectral power analysis is not immune to limitations due to its susceptibility to signal artifacts. EEG data recorded at the scalp level are affected by numerous extraneous sources such as ocular, cardiac, muscle, line, and channel noise. Although various preprocessing techniques exist to mitigate these artifacts, their effectiveness remains inconsistent (Delorme 2023). As EEG signals are known to be non‐stable and non‐linear (Khanna et al. 2015), identifying reliable spectral indices remains a central challenge.

To improve the stability of spectral features, this study evaluates two strategies for expressing spectral power: (1) using relative power values, calculated by dividing the power of each frequency band by total power, and (2) using spectral power ratios, derived by dividing power in one frequency band by that of another (e.g., frontal theta/alpha ratio). The relative power approach minimizes individual differences in overall signal amplitude and mitigates the influence of broadband noise. Spectral power ratios, in turn, capture inter‐band dynamics that may better reflect underlying cortical interactions and are less sensitive to global power fluctuations.

Numerous studies have employed spectral power ratios to quantify cognitive states and detect psychiatric abnormalities. For example, Chang and Choi (2023) demonstrated that the alpha/beta ratio across anterior frontal, frontal, central, parietal, occipital, and temporal regions significantly distinguished patients with depression from healthy controls, with area under the curve (AUC) values significantly higher than 0.5 in receiver operating characteristic (ROC) analyses. Similarly, decreases in the theta/beta ratio in central and parietal regions have also been reported in depressed individuals, reflecting impaired cortical regulation of attention and arousal systems. Importantly, the theta/beta ratio has been validated in attention‐deficit hyperactivity disorder (ADHD) research and is recognized by the United States Food and Drug Administration (FDA) as a supportive diagnostic biomarker when combined with clinical assessment (Stein et al. 2016). This is consistent with another finding that the theta/beta ratio is a good marker for attentional control (Angelidis et al. 2016). Beyond psychiatric disorders, spectral power ratios have also shown discriminative power in neurodegenerative diseases. The theta/alpha ratio, for instance, has consistently differentiated patients with Alzheimer's disease or frontotemporal dementia from healthy older adults across multiple independent studies (Schmidt et al. 2013; Özbek et al. 2021; Chang and Chang 2023), supporting its relevance as a non‐invasive marker of cognitive decline. Also, theta/alpha and theta/beta ratios have been discovered as biomarkers to distinguish Alzheimer's and Lewy body disease (Baik et al. 2022). Temporal beta/gamma ratio also distinguished patients with frontotemporal dementia from healthy controls (Chang and Chang 2023).

Despite these promising findings, no previous studies have systematically assessed the test‐retest reliability of spectral power ratios. Test‐retest reliability is an important characteristic of biomarkers as a within‐individual similarity of repeated measurements across sessions, often measured as an intraclass correlation coefficient that addresses both the degree of correlation and agreement between measurements (Koo and Li 2016). Though some studies have attempted to evaluate the test‐retest reliability of popular measures such as theta/beta ratio and delta/beta ratio (Martin et al. 2025; Anaya et al. 2021), there has been no report of thorough analysis on all possible combinations of spectral power ratios with both absolute and relative values. The current study fills this gap by evaluating the reliability of both absolute and relative spectral power and spectral power ratio measures across two independent datasets. We hypothesize that spectral power ratios will demonstrate superior test‐retest reliability compared to single‐band power measures by mitigating broadband noise activity, and that relative power values will outperform absolute values in terms of stability by normalizing through total frequency band power.

## 2 Methods

### 2.1 Dataset 1

#### 2.1.1 Participants

Dataset 1 was obtained from a publicly available EEG dataset on OpenNeuro originally designed to examine test‐retest reliability across five mental states in healthy adults: eyes‐closed rest, eyes‐open rest, mental arithmetic (subtraction), autobiographical memory recall, and music imagery (Wang et al. 2022). Inclusion criteria were (1) right‐handedness, (2) body mass index (BMI) < 28, and (3) sleep onset prior to 12:30 a.m. on the day before recording. Exclusion criteria included (1) current psychiatric or neurological disorders, (2) use of psychoactive medication within the past three months, and (3) history of head trauma. Participants were instructed to refrain from alcohol and caffeine on the day of EEG recording. A total of 60 healthy adults (mean age = 20 years, SD = 2; 32 female) participated and were compensated (∼$30) for their involvement.

#### 2.1.2 Experimental Procedure

Each participant completed two laboratory visits separated by a one‐month interval. On Visit 1, the first session began with administration of the Self‐rating Anxiety Scale (SAS), Self‐rating Depression Scale (SDS), and Epworth Sleepiness Scale (ESS) (Zung 1965, 1971; Johns 1991). Participants then completed a 5‐min eyes‐open resting EEG, followed by the Mini New York Cognition Questionnaire (mini NYC‐Q; Gorgolewski et al. 2015). This sequence was repeated for 5‐minute eyes‐closed resting EEG. Participants subsequently completed the Amsterdam Resting‐State Questionnaire (ARSQ 2.0), Karolinska Sleepiness Scale (KSS), and Positive and Negative Affect Schedule (PANAS) (Diaz et al. 2014; Akerstedt and Gillberg 1990; Watson et al. 1988). Next, they underwent three EEG recordings during cognitive tasks (subtraction, memory recall, and music imagery), with the mini NYC‐Q administered after each. After a ∼90‐min break, participants completed a second session on the same day following the same protocol, except without SAS, SDS, and ESS. The entire procedure was repeated in full during the second visit.

#### 2.1.3 EEG Acquisition and Preprocessing

Resting‐state EEG recordings were used exclusively for the present analysis, focusing on the eyes‐closed condition due to its widespread application and reduced susceptibility to ocular artifacts. EEG was recorded using either a 63‐ or 64‐channel Ag/AgCl electrode cap (Brain Products GmbH, Germany), based on the extended 10–20 international system. Two channels recorded electrooculograms, and FCz served as the online reference. Data were sampled at 500 Hz, and impedance was maintained below 5 kΩ.

After resampling to 200 Hz, preprocessing was performed using EEGLAB (Delorme and Makeig 2004), with the Artifact Subspace Reconstruction (ASR) plugin. After filtering with 0.5–100 Hz was applied, burst artifacts were removed using a 0.5‐s sliding window and a threshold of 20 standard deviations. Bad data segments were additionally excluded using RMS thresholds (−lnf7) with a 25% channel outlier limit. Following average re‐referencing, independent component analysis (ICA) was applied, and artifact‐related components (e.g., ocular, cardiac, muscle, line noise) were removed using BEM dipfit models with MNI‐based electrode coordinates. Cleaned EEG data were re‐referenced to the average reference.

#### 2.1.4 Spectral Analysis

Spectral power was computed for canonical frequency bands: delta (0.5–4 Hz), theta (4–8 Hz), alpha (8–13 Hz), beta (> 13–30 Hz), and gamma (30–70 Hz). The discrete FFT was applied in a 2.5‐second FFT window length with a frequency resolution of 10 steps per Hz. Spectral power was then averaged within five anatomical regions: frontal (AF3, AF4, AF7, AF8, F1, F2, F3, F4, F5, F6, F7, F8, Fp1, Fp2, Fz), central (C1, C2, C3, C4, C5, C6, Cz, FC1, FC2, FC3, FC4, FC5, FC6), parietal (CP1, CP2, CP3, CP4, CP5, CP6, P1, P2, P3, P4, P5, P6, P7, P8, Pz), occipital (O1, O2, Oz, PO3, PO4, PO7, PO8, POz), and temporal (FT7, FT8, T7, T8, TP8, TP9, TP7, TP10). Spectral power ratios were calculated for each region: delta/theta, delta/alpha, delta/beta, delta/gamma, theta/alpha, theta/beta, theta/gamma, alpha/beta, alpha/gamma, and beta/gamma. For relative spectral power analysis, each ratio was normalized by dividing by the total spectral power across all frequency bands.

### 2.2 Dataset 2

#### 2.2.1 Participants

Dataset 2 was sourced from a separate OpenNeuro dataset (Cavanagh 2021), originally designed to investigate EEG correlates of depression and anxiety (Cavanagh et al. 2019). Participants were university students aged 18–25 years, with no history of seizures or head trauma and no current psychoactive medication use. From the 122 available participants, those with elevated Beck Depression Inventory (BDI) scores (> 9), inconsistent mood assessments, or missing recordings were excluded. The final sample included 74 healthy participants (mean age = 19 years, SD = 1; 39 female) with low, stable BDI scores (< 7) and no reported anxiety disorders.

#### 2.2.2 Experimental Procedure

Each participant underwent two EEG sessions, one prior to and one following a cognitive task (not analyzed here because only one session applied was applied (test‐retest reliability cannot be measured)). In each session, participants completed the Beck Depression Inventory (BDI) and the Spielberger Trait Anxiety Inventory (STAI), followed by 6 min of EEG recording. The resting‐state EEG consisted of 1‐min eyes‐open and 1‐min eyes‐closed segments. Only the eyes‐closed EEG data were analyzed in this study.

#### 2.2.3 EEG Acquisition and Preprocessing

EEG was acquired using a 64‐channel Synamps2 system (Compumedics Neuroscan) with a 10/10 electrode configuration. Data were sampled at 500 Hz with a bandpass filter of 0.5–100 Hz, and impedance was kept below 10 kΩ. The reference electrode was positioned between Cz and CPz. Data were downsampled to 256 Hz, and eyes‐closed segments were extracted. Preprocessing followed the same pipeline as Dataset 1, except for the omission of ASR artifact rejection to preserve segment duration.

#### 2.2.4 Spectral Analysis

The same spectral analysis protocol was applied as the first dataset. Spectral power was averaged differently within five anatomical regions: frontal (AF3, AF4, F1, F2, F3, F4, F5, F6, F7, F8, Fp1, Fp2, Fpz, Fz), central (C1, C2, C3, C4, C5, C6, Cz, FC1, FC2, FC3, FC4, FC5, FC6, FCz), parietal (CP1, CP2, CP3, CP4, CP5, CP6, CPz, P1, P2, P3, P4, P5, P6, P7, P8, Pz), occipital (O1, O2, Oz, PO3, PO4, PO5, PO6, PO7, PO8, POz), and temporal (FT7, FT8, T7, T8, TP8, TP7).

### 2.3 Statistical Analysis

All statistical analyses were conducted using MATLAB R2024b (MathWorks, Natick, MA) and MedCalc Statistical Software v20.218 (MedCalc Software Ltd., Ostend, Belgium).

In Dataset 1, paired *t*‐tests were used to assess differences in SAS, SDS, and ESS scores between Sessions 1 and 3. Repeated measures ANOVA with Huynh‐Feldt correction (*ε* > 0.75) was used for KSS, PANAS, mini NYC‐Q, and ARSQ scores across Sessions 1, 2, and 3 (Girden 1992; Huynh and Feldt 1976). False discovery rate (FDR) correction using the Benjamini–Hochberg procedure was applied to control for multiple comparisons.

Test‐retest reliability of spectral features was assessed using intraclass correlation coefficients (ICC (2, k), two‐way random effects, absolute agreement, average measures), following established guidelines (Koo and Li 2016). ICCs were computed at two levels: (1) per cortical region and (2) per individual electrode. In Dataset 1, ICCs were calculated for three time spans: session 1 versus 2 (short‐term), session 1 versus 3 (long‐term), and across all three sessions (overall reliability). In Dataset 2, only short‐term reliability (Session 1 vs. 2) was assessed. Reliability thresholds followed standard interpretations: ICC ≥ 0.75 was considered “good,” and ICC ≥ 0.90 was “excellent.”

## 3 Results

### 3.1 Stability of Behavioral Scores (Dataset 1)

To evaluate the stability of cognitive and behavioral states across sessions, paired *t*‐tests and repeated measures ANOVA were conducted on behavioral scores in Dataset 1. Paired *t*‐tests showed no significant differences between Session 1 and Session 3 in SAS (*p* = 0.173), SDS (*p* = 0.124), and ESS (*p* = 0.272), indicating no major changes in anxiety, depression, or sleepiness levels over the 1‐month interval (Table 1).

**TABLE 1:** SAS, SDS, and ESS scores between Sessions 1 and 3.

Measure	First session	Third session	*p*‐value
SAS	40.7 ± 8.4	41.9 ± 9.2	0.173
SDS	44.4 ± 8.2	45.9 ± 9.6	0.124
ESS	9.5 ± 3.5	10.1 ± 3.4	0.272

Repeated measures ANOVA with Huynh–Feldt correction, followed by FDR‐adjusted p‐values, revealed no significant session effects across KSS, PANAS (positive/negative affect), ARSQ 2.0 items, or mini NYC‐Q subscales (all adjusted *p* > 0.125; Table 2). These results suggest stable behavioral and cognitive states across all three sessions, supporting the interpretation that any EEG differences likely reflect measurement variability rather than state changes.

**TABLE 2:** KSS, PANAS, mini‐NYC‐Q, ARSQ 2.0, and cognitive task (subtraction, memory, and music) scores between Sessions 1, 2, and 3.

Measure	Session 1	Session 2	Session 3	*p*‐value	adjusted *p*‐value
KSS	5.4 ± 0.2	5.3 ± 0.2	5.4 ± 0.2	0.705	0.757
PANAS_P	30.5 ± 0.8	29.2 ± 1.0	29.1 ± 0.8	0.215	0.443
PANAS_N	20.4 ± 0.9	18.2 ± 0.9	19.4 ± 0.9	0.036	0.261
Discontinuity of mind	6.3 ± 0.4	5.9 ± 0.3	5.8 ± 0.3	0.237	0.443
Theory of mind	6.6 ± 0.4	6.5 ± 0.4	6.0 ± 0.3	0.298	0.443
Self	8.8 ± 0.2	8.4 ± 0.3	8.2 ± 0.3	0.061	0.352
Planning	9.3 ± 0.3	8.4 ± 0.4	8.0 ± 0.3	0.011	0.125
Sleepiness	5.0 ± 0.4	4.4 ± 0.4	4.8 ± 0.4	0.322	0.444
Comfort	7.2 ± 0.3	6.5 ± 0.4	7.5 ± 0.3	0.009	0.125
Somatic awareness	4.5 ± 0.4	4.0 ± 0.4	4.0 ± 0.3	0.216	0.443
Health concern	7.5 ± 0.3	7.3 ± 0.2	7.0 ± 0.2	0.233	0.443
Visual thought	8.8 ± 0.4	9.1 ± 0.3	8.6 ± 0.3	0.486	0.563
Verbal thought	5.4 ± 0.4	4.5 ± 0.4	5.0 ± 0.3	0.075	0.352
Subtraction: Correct/Not correct	0.4 ± 0.1	0.5 ± 0.1	0.5 ± 0.1	0.193	0.443
Subtraction: Final number	4547 ± 35	4376 ± 95	4465 ± 39	0.145	0.443
Memory	1.5 ± 0.1	1.5 ± 0.1	1.7 ± 0.2	0.204	0.443
Music	3.9 ± 0.4	3.3 ± 0.2	3.6 ± 0.4	0.360	0.474
Positive	6.1 ± 0.4	5.8 ± 0.4	5.8 ± 0.4	0.841	0.871
Negative	3.7 ± 0.4	2.8 ± 0.4	2.8 ± 0.4	0.085	0.352
Future	7.4 ± 0.4	5.8 ± 0.5	6.4 ± 0.4	0.013	0.125
Past	6.4 ± 0.4	6.1 ± 0.5	5.6 ± 0.4	0.418	0.527
Myself	7.7 ± 0.4	7.9 ± 0.4	7.2 ± 0.4	0.255	0.443
People	7.6 ± 0.4	7.2 ± 0.4	7.0 ± 0.4	0.281	0.443
Surroundings	5.7 ± 0.4	4.9 ± 0.5	4.8 ± 0.5	0.172	0.443
Vigilance	6.4 ± 0.4	6.9 ± 0.4	6.3 ± 0.4	0.302	0.443
Images	7.4 ± 0.4	7.1 ± 0.4	6.9 ± 0.4	0.552	0.615
Words	3.7 ± 0.4	3.5 ± 0.4	3.7 ± 0.4	0.907	0.907
Specific‐vague	6.7 ± 0.4	6.9 ± 0.3	6.3 ± 0.4	0.306	0.443
Intrusive	4.8 ± 0.4	4.4 ± 0.4	5.0 ± 0.4	0.448	0.541

*Note: p*‐values were adjusted by Benjamini–Hochberg FDR correction for multiple comparisons. “Discontinuity of Mind,” “Theory of Mind, Self,” “Planning,” “Sleepiness,” “Comfort,” “Somatic awareness,” “Health concern,” “Visual thought,” “Verbal thought” are items of ARSQ 2.0, while “Positive,” “Negative,” “Future,” “Past,” “Myself,” “People,” “Surroundings,” “Vigilance,” “Images,” “Words,” “Specific‐vague,” and “Intrusive” are items of mini NYC‐Q.

### 3.2 Test‐Retest Reliability of Spectral Power Features (Dataset 1)

**FIGURE 1:**
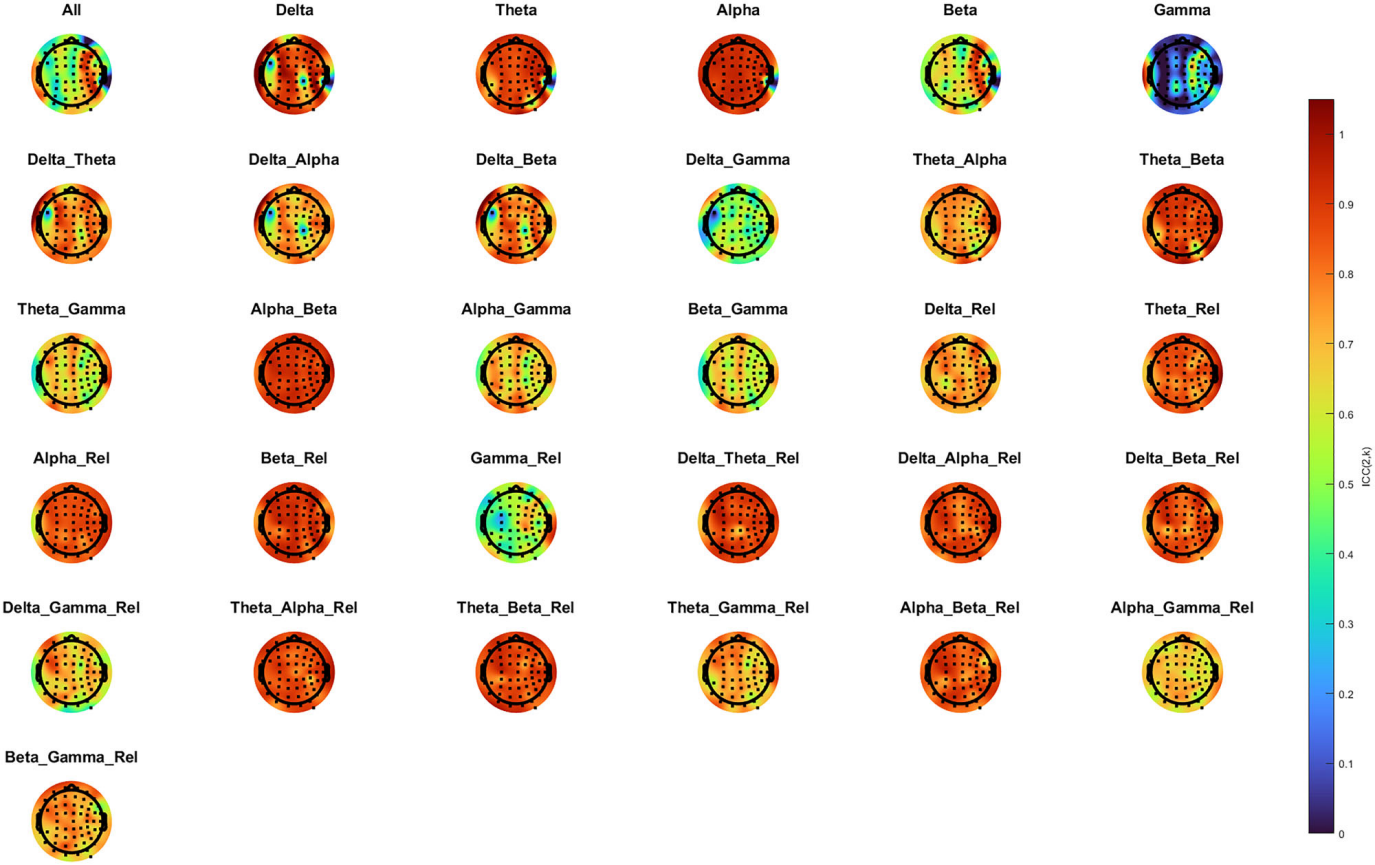
Topographic maps of reliability across spectral power features in Dataset 1. Blue represents low ICCs, and red represents high ICCs. ICCs of relative and absolute single spectral power and relative and absolute spectral power ratios were analyzed.

Intraclass correlation coefficients (ICCs) were computed for each spectral feature at the electrode level. Topographic maps (Figure 1) showed that reliability varied across both spatial regions and frequency bands.

ICC values were also averaged by cortical region (frontal, central, parietal, occipital, temporal). ICCs were calculated in three comparisons: (Session 1 vs. 2 (short‐term), Session 1 versus 3 (long‐term), and Sessions 1–3 (overall stability) in dataset 1 (Supplementary Table 1). F‐alpha, C‐theta, C‐alpha, P‐delta, P‐theta, P‐alpha, O‐theta, C‐alpha/beta, P‐alpha/beta, O‐alpha/beta, T‐alpha/beta, R‐C‐beta, R‐P‐beta, R‐O‐beta, R‐C‐delta/theta, R‐P‐delta/theta, R‐P‐delta/beta, R‐P‐theta/beta, R‐P‐alpha/beta, R‐O‐delta/theta, R‐O‐delta/alpha, R‐T‐delta/theta, R‐T‐delta/alpha, and R‐T‐theta/alpha showed good ICCs (> 0.75) for all three comparisons (Table 3).

**TABLE 3:** Good ICCs (ICC > 0.75) of each spectral power feature across each region in Dataset 1.

Measure	Sessions 1, 2, 3	95% CI	Sessions 1, 2	95% CI	Sessions 1, 3	95% CI
F_Alpha	0.9301	0.8893–0.9568	0.9692	0.9473–0.9818	0.8729	0.7822–0.9251
C_Theta	0.8783	0.8134–0.9232	0.8691	0.7806–0.9219	0.8765	0.7931–0.9263
C_Alpha	0.944	0.9106–0.9655	0.9672	0.9407–0.9813	0.9098	0.8475–0.9464
P_Delta	0.9545	0.9302–0.9713	0.9580	0.9290–0.9750	0.9169	0.8612–0.9503
P_Theta	0.922	0.8796–0.9510	0.9496	0.9121–0.9706	0.8701	0.7832–0.9223
P_Alpha	0.9289	0.8890–0.9557	0.9731	0.9523–0.9845	0.8641	0.7723–0.9188
O_Theta	0.9523	0.9256–0.9702	0.9421	0.8984–0.9664	0.9419	0.9029–0.9653
C_alpha_beta	0.9319	0.8940–0.9575	0.9388	0.8976–0.9635	0.8906	0.8073–0.9366
P_alpha_beta	0.9289	0.8894–0.9556	0.9438	0.8969–0.9682	0.8728	0.7725–0.9269
O_alpha_beta	0.9009	0.8476–0.9376	0.8692	0.7714–0.9238	0.8579	0.7615–0.9153
T_alpha_beta	0.9197	0.8769–0.9493	0.9297	0.8818–0.9581	0.8623	0.7692–0.9178
R_C_Beta	0.9237	0.8831–0.9519	0.9479	0.9106–0.9693	0.8642	0.7727–0.9188
R_P_Beta	0.9445	0.9149–0.9650	0.9765	0.9535–0.9872	0.8987	0.8305–0.9394
R_O_Beta	0.9175	0.8735–0.9480	0.9264	0.8668–0.9580	0.8524	0.7537–0.9116
R_C_delta_theta	0.9386	0.9013–0.9624	0.9286	0.8486–0.9625	0.9125	0.8535–0.9477
R_P_delta_theta	0.9419	0.9081–0.9641	0.9533	0.9188–0.9727	0.9205	0.8656–0.9528
R_P_delta_beta	0.9436	0.8901–0.9690	0.9534	0.9047–0.9751	0.9227	0.8460–0.9581
R_P_theta_beta	0.926	0.8764–0.9557	0.9047	0.8409–0.9430	0.8939	0.7859–0.9429
R_P_alpha_beta	0.9201	0.8772–0.9497	0.8669	0.7753–0.9209	0.9425	0.9036–0.9656
R_O_delta_theta	0.9094	0.8611–0.9429	0.905	0.8384–0.9438	0.9005	0.8332–0.9406
R_O_delta_alpha	0.9253	0.8832–0.9534	0.9575	0.9275–0.9749	0.8743	0.7888–0.9251
R_T_delta_theta	0.9282	0.8866–0.9555	0.9302	0.8707–0.9607	0.893	0.8214–0.9360
R_T_delta_alpha	0.9378	0.8973–0.9626	0.9687	0.9462–0.9816	0.8946	0.8117–0.9394
R_T_theta_alpha	0.9325	0.8955–0.9577	0.9695	0.9489–0.9818	0.8761	0.7880–0.9270

**Abbreviations**: C, central; CI, confidence interval; F, frontal; O, occipital; P, parietal; R, relative; T, temporal.

### 3.3 Test‐Retest Reliability in an Independent Dataset (Dataset 2)

**FIGURE 2:**
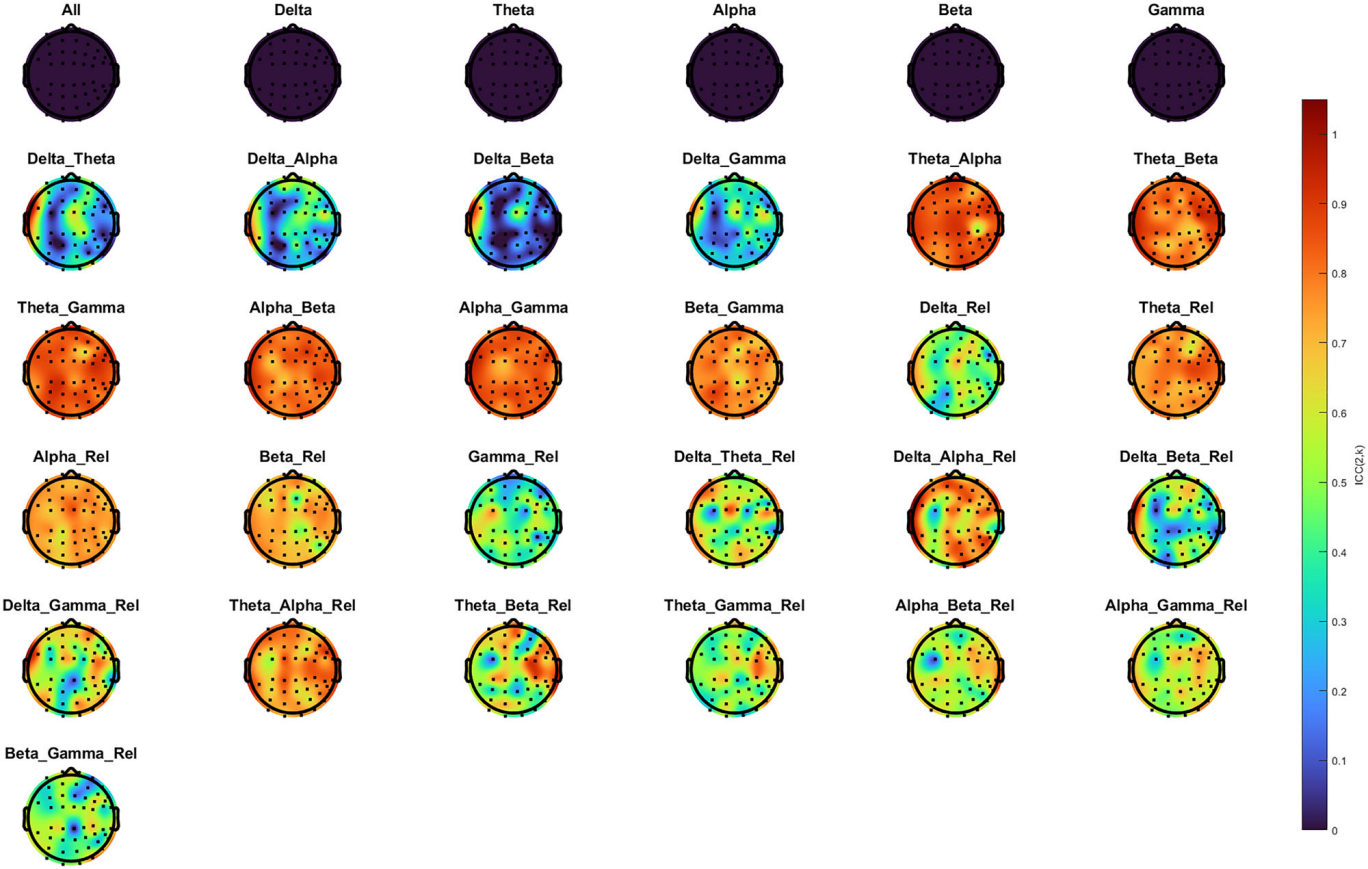
Topographic maps of reliability across spectral power features in Dataset 2. Blue represents low ICCs, and red represents high ICCs. ICCs of relative and absolute single spectral power and relative and absolute spectral power ratios were analyzed.

In Dataset 2, ICCs were computed between the two sessions (short‐term reliability only). In the same way, ICCs across each spectral power feature were measured through individual electrodes (Figure 2). While many absolute and relative power features showed near‐zero or negative ICCs, several spectral power ratios demonstrated robust reliability. Also, ICCs of each spectral power feature across each region were measured between Sessions 1 and 2 in Dataset 2 (Supplementary Table 2). F‐theta‐alpha, F‐theta‐beta, F‐alpha‐beta, F‐alpha‐gamma, C‐theta‐alpha, C‐theta‐gamma, C‐alpha‐beta, C‐alpha‐gamma, C‐beta‐gamma, P‐theta‐alpha, P‐theta‐beta, P‐theta‐gamma, P‐alpha‐beta, P‐alpha‐gamma, O‐theta‐alpha, O‐theta‐beta, O‐theta‐gamma, O‐alpha‐beta, T‐theta‐alpha, T‐theta‐gamma, T‐alpha‐beta, T‐alpha‐gamma, R‐F‐delta‐alpha, R‐P‐theta‐alpha, R‐O‐theta‐alpha, and R‐O‐theta‐beta showed good ICCs (> 0.75) between Sessions 1 and 2 (Table 4).

**TABLE 4:** Good ICCs (ICC > 0.75) of each spectral power feature across each region in Dataset 2.

Measure	Sessions 1, 2	95% CI
F_theta_alpha	0.8844	0.8169–0.9271
F_theta_beta	0.8509	0.7622–0.9063
F_alpha_beta	0.9008	0.8424–0.9376
F_alpha_gamma	0.9103	0.8574–0.9435
C_theta_alpha	0.9005	0.8424–0.9373
C_theta_gamma	0.8957	0.8348–0.9342
C_alpha_beta	0.8606	0.7790–0.9121
C_alpha_gamma	0.9083	0.8547–0.9422
C_beta_gamma	0.8564	0.7722–0.9095
P_theta_alpha	0.8901	0.8259–0.9307
P_theta_beta	0.8619	0.7799–0.9132
P_theta_gamma	0.9028	0.8460–0.9387
P_alpha_beta	0.8661	0.7872–0.9157
P_alpha_gamma	0.9002	0.8406–0.9374
O_theta_alpha	0.8449	0.7525–0.9026
O_theta_beta	0.912	0.8557–0.9457
O_theta_gamma	0.9034	0.8465–0.9391
O_alpha_beta	0.8616	0.7807–0.9127
T_theta_alpha	0.9063	0.8492–0.9415
T_theta_beta	0.7818	0.6545–0.8623
T_theta_gamma	0.898	0.8384–0.9357
T_alpha_beta	0.8606	0.7785–0.9123
T_alpha_gamma	0.903	0.8460–0.9389
R_F_delta_alpha	0.8566	0.7724–0.9096
R_P_theta_alpha	0.8512	0.7637–0.9063
R_O_theta_alpha	0.8662	0.7874–0.9157
R_O_theta_beta	0.8615	0.7801–0.9128

**Abbreviations**: C, central; CI, confidence interval; F, frontal; O, occipital; P, parietal; R, relative; T, temporal.

By comparing results across both datasets, C‐alpha/beta, P‐alpha/beta, O‐alpha/beta, and T‐alpha/beta consistently showed good ICCs (> 0.75) for all measurements.

## 4 Discussion

### 4.1 Test‐Retest Reliability of Spectral Power Features

The present study assessed the test‐retest reliability of various spectral EEG features, including absolute and relative single‐band power and spectral power ratios, across two independent datasets. Dataset 1 incorporated both short‐term (within‐day) and long‐term (1‐month interval) comparisons, while Dataset 2 provided an independent replication with a shorter interval. ICCs of each spectral power measure were calculated, resulting in various reliability scores from negative (which represents an inappropriate biomarker, as intra‐individual difference exceeds inter‐individual difference) to positive values. Across all analyses, spectral power ratios demonstrated higher reliability than single‐band spectral power, consistent with our primary hypothesis.

In Dataset 1, behavioral scores remained statistically stable across sessions, confirming that EEG differences were unlikely to result from cognitive or emotional fluctuations. ICC analyses showed that several ratio features, especially absolute alpha/beta ratios in central (C), parietal (P), occipital (O), and temporal (T) regions, consistently yielded good reliability (ICC > 0.75) across all timepoints. This pattern was replicated in Dataset 2, despite differences in acquisition systems, preprocessing, and participant population.

These findings support that spectral power ratios, particularly the absolute alpha/beta ratio, are more stable over time than traditional single‐band metrics. The higher reliability of ratios likely stems from their ability to normalize inter‐individual variability and suppress non‐neural artifacts. As both numerator and denominator are drawn from the same EEG signal, shared sources of noise (e.g., movement, impedance variation, general arousal) may be reduced through cancellation, resulting in a more stable metric across time. Furthermore, the robustness across spatial regions suggests that the alpha/beta ratio is not restricted to a single functional network but instead reflects a distributed neural property, potentially related to cortical excitability and cognitive control. Possible explanations for why the “absolute” alpha/beta ratio is more reliable than the relative alpha/beta ratio could be due to the introduction of noise or an unnecessary signal from total frequency band (e.g., 1/f background activity) in the process of normalization in the relative power calculation (Gyurkovics et al., 2021). This might be inconsistent with our second hypothesis that relative power would be more reliable than absolute power. Also, this might explain why other ratios like the delta/theta ratio were discovered to be unreliable because 1/f activity increases as frequency decreases.

Also, the second dataset has its own limitation in that there was no cognitive test to ensure that cognitive states were consistent before and after the cognitive task, as in the first dataset. Therefore, although the absolute alpha/beta ratio has its own value as a reliable measure across two independent datasets, other spectral power measures with high test‐retest reliability for only the first dataset (e.g., relative central beta power) are also valuable markers as EEG measures and further investigations are needed.

### 4.2 Clinical and Cognitive Relevance of the Alpha/Beta Ratio

Although this is the first study to directly evaluate the test‐retest reliability of spectral power ratios, the alpha/beta ratio has already been widely used in both cognitive and clinical EEG research. For instance, Chang and Choi (2023) demonstrated that reduced alpha/beta ratios across frontal, central, and parietal regions were significantly associated with depression when compared to healthy controls, with AUC values exceeding 0.7 in ROC curves.

Frontal and parietal alpha/beta showed significant negative correlation with pain scores of patients with lumbar disk herniation (Li et al. 2025). Also, frontal alpha/beta showed significant differences (*p*‐value < 0.05) between patients with frontotemporal dementia and healthy controls (Chang and Chang 2023). In healthy individuals, the alpha/beta ratio was significantly related to stress (Yi and Mohd 2020). Collectively, these studies highlight the potential of the alpha/beta ratio not only as a state‐sensitive index of cortical functioning but also as a reliable biomarker for longitudinal or diagnostic applications. The present study adds an essential dimension to this body of evidence by establishing that alpha/beta ratios can be measured reproducibly over both short‐ and long‐term intervals, supporting their use in clinical monitoring and treatment evaluation.

### 4.3 Comparison With Previous Reports

Many previously published studies on test‐retest reliability of spectral power have discovered that spectral power in relative and absolute value is highly reliable (Gudmundsson et al. 2007; Tenke et al. 2018; Getzmann et al. 2024; Metzen et al. 2022). However, each result of these studies is dependent on an individual recording system and preprocessing pipeline. In the current study, within each dataset, many measures of spectral power showed good test‐retest reliability (ICC > 0.75) in the short term and the long term, which is consistent with the results from previous studies. However, in comparison with two independent datasets, only the alpha/beta ratio exhibited consistent test‐retest reliability. This might indicate the importance of standardized processing procedures in reliability studies, as EEG has high variability with preprocessing and analytic procedures (Delorme 2023). Also, many previous studies on test‐retest reliability did not include extensive cognitive or behavioral tests such as Dataset 1 to regulate possible changes in cognitive states over time (Gudmundsson et al. 2007; Tenke et al. 2018; Getzmann et al. 2024; Metzen et al. 2022). This limitation in previous reports indicates that observed spectral power measures in those studies might be affected by intra‐individual cognitive or behavioral changes over time.

### 4.4 Limitations

One of the strengths of this study is the use of two independent EEG datasets with different experimental protocols, recording hardware, and preprocessing pipelines. The replication of key findings across these datasets increases confidence in the generalizability and robustness of spectral ratio reliability. The consistency of alpha/beta ratios in particular suggests that this feature is less sensitive to methodological variation than many traditional EEG metrics.

However, several limitations should be acknowledged. First, while Dataset 1 included extensive behavioral assessments confirming stable cognitive states across sessions, Dataset 2 lacked such measures. As a result, we cannot fully exclude the possibility of unmeasured cognitive variation contributing to spectral fluctuations in Dataset 2. Nonetheless, the short time interval between sessions (approximately 1 h) reduces the likelihood of significant state changes.

Second, although preprocessing was kept consistent across datasets where possible, the choice of preprocessing pipeline can strongly affect spectral power values (Delorme 2023). A previous report has discovered that preprocessing, such as automatic data corrections, could lead to loss of statistical power and performance (Delorme 2023). Nevertheless, adequate preprocessing is theoretically necessary to remove noise from non‐brain sources (e.g., heart, eye muscle) and facilitate signal processing (Delorme and Makeig 2004). Future studies should systematically assess how different artifact rejection techniques (e.g., ICA variants, ASR thresholds), referencing schemes, and frequency decomposition methods affect the reliability of spectral features. The high reliability of spectral ratios observed here may not generalize to all analytic pipelines.

Also, Dataset 2 was not cleaned with ASR artifact rejection due to short segment duration. Thus, Dataset 2 is more vulnerable to noise despite further noise removal by ICA. This might lead to inconsistency in test‐retest reliability between Datasets 1 and 2.

Finally, while ICC provides a robust measure of test‐retest reliability, it does not capture sensitivity to cognitive or clinical change. While certain spectral power ratios could be highly reliable, if those ratios could not distinguish cognitive or clinical states, the usefulness of those markers is limited. That is, while more unstable theta/beta or theta/alpha ratios have been published as a probable biomarker for many clinical abnormalities, including dementia or ADHD (Stein et al. 2016; Chang and Chang 2023), alpha/beta is relatively limited in its clinical significance for depression or stress (Chang and Choi 2023). Thus, future work should evaluate both reliability and discriminability—the ability of features to distinguish meaningful cognitive or clinical states—in parallel.

## 5 Conclusions

This study is the first to systematically evaluate the test‐retest reliability of EEG spectral power ratios alongside absolute and relative single‐band power across both short‐term and long‐term intervals. Using two independent datasets of healthy individuals, we demonstrate that the alpha/beta ratio, particularly in central, parietal, occipital, and temporal regions, exhibits consistently high reliability (ICC > 0.75) regardless of time interval or dataset origin.

Compared to traditional spectral power metrics, spectral power ratios provided a more robust and stable measure across sessions. This suggests that ratio‐based indices may help mitigate noise and inter‐individual variability, making them especially well‐suited for clinical and cognitive applications where reproducibility is critical.

The findings support the use of spectral power ratios, especially the alpha/beta ratio, as promising biomarkers for longitudinal EEG studies and as candidate features in the development of diagnostic tools for neurological and psychiatric conditions. Future work should expand on this foundation by exploring how preprocessing choices affect reliability and by assessing these features in clinical populations where tracking changes over time is essential.

## Author Contributions

The corresponding author contributes to the study.

## Funding

The author has nothing to report.

## Conflicts of Interest

The author declares no conflicts of interest.

## Peer Review

The peer review history for this article is available at https://doi.org/10.1002/brb3.71035

## Supporting information




**Supplementary Tables**: brb371035‐sup‐0001‐TableS1‐S2.docx

## Data Availability

The original dataset for first dataset could be found in OpenNeuro: https://doi.org/10.18112/openneuro.ds004148.v1.0.1 The original dataset for second dataset could be found in OpenNeuro: https://doi.org/10.18112/openneuro.ds003478.v1.1.0
