# Supplementary material for: Test‐Retest Reliability of Single Spectral Power and Spectral Power Ratios in Relative and Absolute Values
Source: Brain Behav. 2025 Nov 5;15(11):e71035. doi: 10.1002/brb3.71035 (PMC12589806; doi:10.1002/brb3.71035)
Supplement: Supplementary file 1 — Supplementary Tables: brb371035‐sup‐0001‐TableS1‐S2.docx [file BRB3-15-e71035-s001.docx]

Supplementary Table 1. ICCs of each spectral power feature across each region in dataset 1

|  | session 1, 2, 3 | | 95% CI | session 1, 2 | | 95% CI | session 1, 3 | | 95% CI |
| --- | --- | --- | --- | --- | --- | --- | --- | --- | --- |
| F_Delta | 0.7041 | 0.5467 - 0.8134 | | 0.8487 | 0.7453 - 0.9099 | | 0.2768 | -0.2139 - 0.5687 | |
| F_Theta | 0.7547 | 0.6230 - 0.8456 | | 0.9005 | 0.8334 - 0.9406 | | 0.4617 | 0.09486 - 0.6793 | |
| F_Alpha | 0.9301 | 0.8893 - 0.9568 | | 0.9692 | 0.9473 - 0.9818 | | 0.8729 | 0.7822 - 0.9251 | |
| F_Beta | 0.552 | 0.3208 - 0.7153 | | 0.4764 | 0.1392 - 0.6838 | | 0.8332 | 0.7195 - 0.9006 | |
| F_Gamma | 0.3652 | 0.03117 - 0.5985 | | 0.5697 | 0.2844 - 0.7420 | | -0.1997 | -1.0171 - 0.2852 | |
| F_All | 0.6854 | 0.5185 - 0.8014 | | 0.7443 | 0.5713 - 0.8474 | | 0.5396 | 0.2351 - 0.7237 | |
| C_Delta | 0.8461 | 0.7642 - 0.9029 | | 0.8432 | 0.7367 - 0.9065 | | 0.6922 | 0.4834 - 0.8164 | |
| C_Theta | 0.8783 | 0.8134 - 0.9232 | | 0.8691 | 0.7806 - 0.9219 | | 0.8765 | 0.7931 - 0.9263 | |
| C_Alpha | 0.944 | 0.9106 - 0.9655 | | 0.9672 | 0.9407 - 0.9813 | | 0.9098 | 0.8475 - 0.9464 | |
| C_Beta | 0.548 | 0.3107 - 0.7140 | | 0.4116 | 0.02534 - 0.6463 | | 0.8791 | 0.7978 - 0.9278 | |
| C_Gamma | 0.01608 | -0.5087 - 0.3797 | | 0.03248 | -0.6169 - 0.4215 | | 0.2797 | -0.2157 - 0.5718 | |
| C_All | 0.3728 | 0.04101 - 0.6038 | | 0.2649 | -0.2260 - 0.5599 | | 0.9331 | 0.8875 - 0.9601 | |
| P_Delta | 0.9545 | 0.9302 - 0.9713 | | 0.9580 | 0.9290 - 0.9750 | | 0.9169 | 0.8612 - 0.9503 | |
| P_Theta | 0.922 | 0.8796 - 0.9510 | | 0.9496 | 0.9121 - 0.9706 | | 0.8701 | 0.7832 - 0.9223 | |
| P_Alpha | 0.9289 | 0.8890 - 0.9557 | | 0.9731 | 0.9523 - 0.9845 | | 0.8641 | 0.7723 - 0.9188 | |
| P_Beta | 0.8516 | 0.7621 - 0.9090 | | 0.8368 | 0.7056 - 0.9067 | | 0.8807 | 0.7996 - 0.9289 | |
| P_Gamma | 0.07667 | -0.4014 - 0.4137 | | 0.1009 | -0.4836 - 0.4582 | | 0.3876 | -0.02699 - 0.6346 | |
| P_All | 0.6389 | 0.4497 - 0.7713 | | 0.5495 | 0.2551 - 0.7288 | | 0.8947 | 0.8221 - 0.9375 | |
| O_Delta | 0.8482 | 0.7673 - 0.9043 | | 0.9469 | 0.9111 - 0.9683 | | 0.7466 | 0.5755 - 0.8487 | |
| O_Theta | 0.9523 | 0.9256 - 0.9702 | | 0.9421 | 0.8984 - 0.9664 | | 0.9419 | 0.9029 - 0.9653 | |
| O_Alpha | 0.925 | 0.8818 - 0.9535 | | 0.9762 | 0.9515 - 0.9872 | | 0.8325 | 0.7202 - 0.8998 | |
| O_Beta | 0.894 | 0.8268 - 0.9358 | | 0.883 | 0.7323 - 0.9409 | | 0.856 | 0.7586 - 0.9141 | |
| O_Gamma | 0.6483 | 0.4635 - 0.7773 | | 0.6271 | 0.3743 - 0.7775 | | 0.7662 | 0.6080 - 0.8604 | |
| O_All | 0.888 | 0.8235 - 0.9306 | | 0.9058 | 0.8179 - 0.9482 | | 0.8476 | 0.7454 - 0.9089 | |
| T_Delta | 0.002526 | -0.5324 - 0.3719 | | 0.004488 | -0.6677 - 0.4056 | | 0.7627 | 0.6025 - 0.8583 | |
| T_Theta | -0.001221 | -0.5371 - 0.3693 | | -0.0008405 | -0.6754 - 0.4021 | | 0.9157 | 0.8588 - 0.9497 | |
| T_Alpha | -0.006963 | -0.5453 - 0.3655 | | -0.003873 | -0.6800 - 0.4002 | | 0.8923 | 0.8200 - 0.9356 | |
| T_Beta | 0.0004930 | -0.5345 - 0.3704 | | -0.0001088 | -0.6743 - 0.4026 | | 0.7290 | 0.5481 - 0.8378 | |
| T_Gamma | 0.0003264 | -0.5348 - 0.3703 | | 0.0004481 | -0.6734 - 0.4029 | | 0.2792 | -0.2065 - 0.5694 | |
| T_All | 0.0001063 | -0.5351 - 0.3701 | | 0.0003119 | -0.6736 - 0.4029 | | 0.8742 | 0.7900 - 0.9247 | |
| F_delta_theta | 0.7615 | 0.6336 - 0.8498 | | 0.8519 | 0.7157 - 0.9182 | | 0.6077 | 0.3426 - 0.7658 | |
| F_delta_alpha | 0.5666 | 0.3413 - 0.7248 | | 0.8342 | 0.6931 - 0.9066 | | 0.2939 | -0.1542 - 0.5721 | |
| F_delta_beta | 0.6458 | 0.4597 - 0.7759 | | 0.817 | 0.6789 - 0.8937 | | 0.2400 | -0.2835 - 0.5484 | |
| F_delta_gamma | 0.6584 | 0.4758 - 0.7847 | | 0.7563 | 0.5872 - 0.8555 | | 0.0242 | -0.6518 - 0.4209 | |
| F_theta_alpha | 0.6088 | 0.4044 - 0.7520 | | 0.8927 | 0.8209 - 0.9358 | | 0.4129 | 0.04122 - 0.6440 | |
| F_theta_beta | 0.7537 | 0.6232 - 0.8444 | | 0.8918 | 0.8027 - 0.9385 | | 0.4974 | 0.1541 - 0.7007 | |
| F_theta_gamma | 0.558 | 0.3262 - 0.7202 | | 0.8322 | 0.6850 - 0.9061 | | 0.2493 | -0.2641 - 0.5532 | |
| F_alpha_beta | 0.8932 | 0.8357 - 0.9328 | | 0.8989 | 0.8220 - 0.9414 | | 0.8531 | 0.7490 - 0.9133 | |
| F_alpha_gamma | 0.6172 | 0.4141 - 0.7583 | | 0.887 | 0.8011 - 0.9345 | | 0.1215 | -0.4641 - 0.4739 | |
| F_beta_gamma | 0.5057 | 0.2438 - 0.6879 | | 0.8995 | 0.8186 - 0.9426 | | -0.1251 | -0.9080 - 0.3330 | |
| C_delta_theta | 0.735 | 0.5908 - 0.8336 | | 0.6544 | 0.3877 - 0.8005 | | 0.5802 | 0.2944 - 0.7499 | |
| C_delta_alpha | 0.6184 | 0.4160 - 0.7589 | | 0.598 | 0.3339 - 0.7584 | | 0.4663 | 0.1221 - 0.6778 | |
| C_delta_beta | 0.7847 | 0.6634 - 0.8659 | | 0.7745 | 0.5935 - 0.8710 | | 0.5992 | 0.3287 - 0.7607 | |
| C_delta_gamma | 0.6052 | 0.3920 - 0.7518 | | 0.7771 | 0.6261 - 0.8670 | | 0.2474 | -0.2703 - 0.5527 | |
| C_theta_alpha | 0.7044 | 0.5419 - 0.8147 | | 0.9157 | 0.8593 - 0.9496 | | 0.5742 | 0.2895 - 0.7451 | |
| C_theta_beta | 0.9155 | 0.8704 - 0.9467 | | 0.9681 | 0.9467 - 0.9809 | | 0.8262 | 0.7100 - 0.8959 | |
| C_theta_gamma | 0.7636 | 0.6374 - 0.8510 | | 0.8516 | 0.7521 - 0.9112 | | 0.5745 | 0.2847 - 0.7465 | |
| C_alpha_beta | 0.9319 | 0.8940 - 0.9575 | | 0.9388 | 0.8976 - 0.9635 | | 0.8906 | 0.8073 - 0.9366 | |
| C_alpha_gamma | 0.7958 | 0.6874 - 0.8711 | | 0.8369 | 0.7278 - 0.9024 | | 0.6694 | 0.4494 - 0.8019 | |
| C_beta_gamma | 0.7662 | 0.6411 - 0.8527 | | 0.8938 | 0.8227 - 0.9364 | | 0.4878 | 0.1379 - 0.6950 | |
| P_delta_theta | 0.8929 | 0.8261 - 0.9348 | | 0.8921 | 0.6896 - 0.9509 | | 0.8311 | 0.7170 - 0.8992 | |
| P_delta_alpha | 0.742 | 0.5908 - 0.8407 | | 0.9439 | 0.8839 - 0.9701 | | 0.6423 | 0.3933 - 0.7880 | |
| P_delta_beta | 0.8968 | 0.8129 - 0.9411 | | 0.9101 | 0.6916 - 0.9619 | | 0.8771 | 0.7943 - 0.9265 | |
| P_delta_gamma | 0.5626 | 0.3365 - 0.7220 | | 0.7594 | 0.4930 - 0.8739 | | 0.3458 | -0.1024 - 0.6108 | |
| P_theta_alpha | 0.689 | 0.5200 - 0.8045 | | 0.9441 | 0.9065 - 0.9666 | | 0.5718 | 0.2863 - 0.7436 | |
| P_theta_beta | 0.9238 | 0.8823 - 0.9522 | | 0.9696 | 0.9454 - 0.9826 | | 0.839 | 0.7313 - 0.9036 | |
| P_theta_gamma | 0.6682 | 0.4933 - 0.7903 | | 0.8593 | 0.7276 - 0.9226 | | 0.3858 | -0.03180 - 0.6339 | |
| P_alpha_beta | 0.9289 | 0.8894 - 0.9556 | | 0.9438 | 0.8969 - 0.9682 | | 0.8728 | 0.7725 - 0.9269 | |
| P_alpha_gamma | 0.7224 | 0.5755 - 0.8247 | | 0.8389 | 0.7227 - 0.9053 | | 0.5286 | 0.2200 - 0.7164 | |
| P_beta_gamma | 0.6875 | 0.5226 - 0.8024 | | 0.8443 | 0.7082 - 0.9128 | | 0.4213 | 0.03378 - 0.6538 | |
| O_delta_theta | 0.69 | 0.5266 - 0.8040 | | 0.87 | 0.7047 - 0.9342 | | 0.5842 | 0.3042 - 0.7516 | |
| O_delta_alpha | 0.6326 | 0.4382 - 0.7677 | | 0.9174 | 0.8484 - 0.9532 | | 0.5401 | 0.2413 - 0.7228 | |
| O_delta_beta | 0.7815 | 0.6552 - 0.8646 | | 0.8411 | 0.6090 - 0.9227 | | 0.7109 | 0.5178 - 0.8270 | |
| O_delta_gamma | 0.5468 | 0.3106 - 0.7126 | | 0.407 | 0.03080 - 0.6406 | | 0.5242 | 0.1992 - 0.7167 | |
| O_theta_alpha | 0.6545 | 0.4731 - 0.7813 | | 0.9281 | 0.8797 - 0.9571 | | 0.4933 | 0.1666 - 0.6940 | |
| O_theta_beta | 0.8692 | 0.7994 - 0.9175 | | 0.9365 | 0.8879 - 0.9632 | | 0.7266 | 0.5417 - 0.8368 | |
| O_theta_gamma | 0.5401 | 0.2930 - 0.7106 | | 0.4861 | 0.1409 - 0.6928 | | 0.4957 | 0.1527 - 0.6994 | |
| O_alpha_beta | 0.9009 | 0.8476 - 0.9376 | | 0.8692 | 0.7714 - 0.9238 | | 0.8579 | 0.7615 - 0.9153 | |
| O_alpha_gamma | 0.4695 | 0.1825 - 0.6667 | | 0.2576 | -0.2522 - 0.5585 | | 0.7212 | 0.5351 - 0.8331 | |
| O_beta_gamma | 0.5719 | 0.3423 - 0.7304 | | 0.4917 | 0.1507 - 0.6960 | | 0.5802 | 0.2984 - 0.7490 | |
| T_delta_theta | 0.8172 | 0.6999 - 0.8895 | | 0.7849 | 0.5409 - 0.8879 | | 0.7595 | 0.5989 - 0.8560 | |
| T_delta_alpha | 0.8059 | 0.6793 - 0.8831 | | 0.8919 | 0.7970 - 0.9395 | | 0.7178 | 0.5125 - 0.8345 | |
| T_delta_beta | 0.8102 | 0.7081 - 0.8806 | | 0.8763 | 0.7910 - 0.9265 | | 0.6742 | 0.4575 - 0.8047 | |
| T_delta_gamma | 0.6584 | 0.4758 - 0.7847 | | 0.806 | 0.6757 - 0.8840 | | 0.3238 | -0.1273 - 0.5950 | |
| T_theta_alpha | 0.8477 | 0.7625 - 0.9050 | | 0.941 | 0.9011 - 0.9647 | | 0.7586 | 0.5846 - 0.8581 | |
| T_theta_beta | 0.9113 | 0.8639 - 0.9441 | | 0.9587 | 0.9310 - 0.9753 | | 0.8329 | 0.7211 - 0.9000 | |
| T_theta_gamma | 0.7435 | 0.6074 - 0.8381 | | 0.8564 | 0.7533 - 0.9156 | | 0.5153 | 0.1862 - 0.7110 | |
| T_alpha_beta | 0.9197 | 0.8769 - 0.9493 | | 0.9297 | 0.8818 - 0.9581 | | 0.8623 | 0.7692 - 0.9178 | |
| T_alpha_gamma | 0.7407 | 0.6032 - 0.8363 | | 0.8077 | 0.6670 - 0.8874 | | 0.5152 | 0.1892 - 0.7102 | |
| T_beta_gamma | 0.648 | 0.4607 - 0.7779 | | 0.8556 | 0.7477 - 0.9159 | | 0.2173 | -0.3158 - 0.5336 | |
| R_F_Delta | 0.7212 | 0.5648 - 0.8261 | | 0.9174 | 0.7767 - 0.9613 | | 0.4362 | 0.06795 - 0.6606 | |
| R_F_Theta | 0.8007 | 0.6940 - 0.8745 | | 0.9475 | 0.9121 - 0.9687 | | 0.5718 | 0.2831 - 0.7443 | |
| R_F_Alpha | 0.7406 | 0.6026 - 0.8364 | | 0.939 | 0.8980 - 0.9636 | | 0.5097 | 0.1819 - 0.7065 | |
| R_F_Beta | 0.8422 | 0.7522 - 0.9020 | | 0.9237 | 0.6592 - 0.9706 | | 0.7089 | 0.5113 - 0.8264 | |
| R_F_Gamma | 0.4915 | 0.2256 - 0.6779 | | 0.9598 | 0.9049 - 0.9800 | | -0.4668 | -1.4744 - 0.1278 | |
| R_C_Delta | 0.6971 | 0.5183 - 0.8133 | | 0.7473 | 0.5076 - 0.8617 | | 0.5272 | 0.2163 - 0.7159 | |
| R_C_Theta | 0.8672 | 0.7963 - 0.9163 | | 0.9312 | 0.8847 - 0.9589 | | 0.7731 | 0.6197 - 0.8646 | |
| R_C_Alpha | 0.8799 | 0.8099 - 0.9258 | | 0.9029 | 0.8378 - 0.9419 | | 0.8233 | 0.6938 - 0.8966 | |
| R_C_Beta | 0.9237 | 0.8831 - 0.9519 | | 0.9479 | 0.9106 - 0.9693 | | 0.8642 | 0.7727 - 0.9188 | |
| R_C_Gamma | 0.5064 | 0.2439 - 0.6886 | | 0.7058 | 0.5105 - 0.8236 | | 0.1265 | -0.4770 - 0.4813 | |
| R_P_Delta | 0.8241 | 0.6762 - 0.9006 | | 0.876 | 0.6144 - 0.9455 | | 0.7734 | 0.6085 - 0.8672 | |
| R_P_Theta | 0.8478 | 0.7636 - 0.9048 | | 0.9226 | 0.8707 - 0.9537 | | 0.751 | 0.5801 - 0.8519 | |
| R_P_Alpha | 0.853 | 0.7736 - 0.9076 | | 0.8838 | 0.8060 - 0.9305 | | 0.7847 | 0.6261 - 0.8742 | |
| R_P_Beta | 0.9445 | 0.9149 - 0.9650 | | 0.9765 | 0.9535 - 0.9872 | | 0.8987 | 0.8305 - 0.9394 | |
| R_P_Gamma | 0.4886 | 0.2261 - 0.6745 | | 0.6601 | 0.4185 - 0.7996 | | 0.1946 | -0.3581 - 0.5210 | |
| R_O_Delta | 0.744 | 0.5944 - 0.8418 | | 0.8112 | 0.5837 - 0.9032 | | 0.7067 | 0.5120 - 0.8242 | |
| R_O_Theta | 0.7877 | 0.6749 - 0.8660 | | 0.9228 | 0.8697 - 0.9541 | | 0.6034 | 0.3359 - 0.7631 | |
| R_O_Alpha | 0.8794 | 0.8146 - 0.9241 | | 0.9155 | 0.8580 - 0.9496 | | 0.7953 | 0.6516 - 0.8789 | |
| R_O_Beta | 0.9175 | 0.8735 - 0.9480 | | 0.9264 | 0.8668 - 0.9580 | | 0.8524 | 0.7537 - 0.9116 | |
| R_O_Gamma | 0.7816 | 0.6645 - 0.8624 | | 0.8286 | 0.6597 - 0.9070 | | 0.6697 | 0.4485 - 0.8023 | |
| R_T_Delta | 0.7467 | 0.6058 - 0.8417 | | 0.8409 | 0.7098 - 0.9095 | | 0.6078 | 0.3492 - 0.7645 | |
| R_T_Theta | 0.9048 | 0.8540 - 0.9400 | | 0.9462 | 0.9098 - 0.9679 | | 0.8092 | 0.6809 - 0.8860 | |
| R_T_Alpha | 0.881 | 0.8145 - 0.9257 | | 0.915 | 0.8460 - 0.9515 | | 0.8228 | 0.7044 - 0.8939 | |
| R_T_Beta | 0.8656 | 0.7937 - 0.9154 | | 0.9061 | 0.8428 - 0.9439 | | 0.755 | 0.5891 - 0.8538 | |
| R_T_Gamma | 0.7151 | 0.5622 - 0.8207 | | 0.8747 | 0.7900 - 0.9252 | | 0.4731 | 0.1151 - 0.6859 | |
| R_F_delta_theta | 0.8735 | 0.8029 - 0.9210 | | 0.9005 | 0.8324 - 0.9408 | | 0.8029 | 0.6702 - 0.8822 | |
| R_F_delta_alpha | 0.8895 | 0.8054 - 0.9360 | | 0.9396 | 0.8974 - 0.9642 | | 0.8456 | 0.6846 - 0.9175 | |
| R_F_delta_beta | 0.8323 | 0.7270 - 0.8981 | | 0.8717 | 0.7481 - 0.9300 | | 0.7909 | 0.6485 - 0.8754 | |
| R_F_delta_gamma | 0.5979 | 0.3852 - 0.7457 | | 0.7304 | 0.4872 - 0.8505 | | 0.5344 | 0.2270 - 0.7204 | |
| R_F_theta_alpha | 0.883 | 0.8163 - 0.9274 | | 0.9367 | 0.8941 - 0.9622 | | 0.8112 | 0.6737 - 0.8893 | |
| R_F_theta_beta | 0.8828 | 0.8190 - 0.9264 | | 0.9051 | 0.8347 - 0.9446 | | 0.7966 | 0.6601 - 0.8784 | |
| R_F_theta_gamma | 0.7346 | 0.5924 - 0.8327 | | 0.837 | 0.6537 - 0.9147 | | 0.5905 | 0.3121 - 0.7559 | |
| R_F_alpha_beta | 0.8186 | 0.7222 - 0.8856 | | 0.8471 | 0.7329 - 0.9110 | | 0.7317 | 0.5525 - 0.8393 | |
| R_F_alpha_gamma | 0.6371 | 0.4468 - 0.7702 | | 0.7873 | 0.6062 - 0.8802 | | 0.3793 | -0.03586 - 0.6286 | |
| R_F_beta_gamma | 0.7445 | 0.6092 - 0.8386 | | 0.8588 | 0.7534 - 0.9178 | | 0.5390 | 0.2265 - 0.7250 | |
| R_C_delta_theta | 0.9386 | 0.9013 - 0.9624 | | 0.9286 | 0.8486 - 0.9625 | | 0.9125 | 0.8535 - 0.9477 | |
| R_C_delta_alpha | 0.8454 | 0.7431 - 0.9072 | | 0.8929 | 0.8095 - 0.9383 | | 0.7494 | 0.5689 - 0.8527 | |
| R_C_delta_beta | 0.8887 | 0.8167 - 0.9329 | | 0.8762 | 0.7821 - 0.9282 | | 0.8227 | 0.7002 - 0.8947 | |
| R_C_delta_gamma | 0.763 | 0.6371 - 0.8504 | | 0.7866 | 0.6421 - 0.8727 | | 0.5597 | 0.2684 - 0.7358 | |
| R_C_theta_alpha | 0.8619 | 0.7705 - 0.9171 | | 0.9415 | 0.9023 - 0.9650 | | 0.7589 | 0.5668 - 0.8618 | |
| R_C_theta_beta | 0.8974 | 0.8402 - 0.9359 | | 0.9261 | 0.8764 - 0.9558 | | 0.803 | 0.6657 - 0.8833 | |
| R_C_theta_gamma | 0.7917 | 0.6808 - 0.8686 | | 0.8373 | 0.7286 - 0.9026 | | 0.5889 | 0.3175 - 0.7533 | |
| R_C_alpha_beta | 0.8723 | 0.8042 - 0.9195 | | 0.8391 | 0.7301 - 0.9040 | | 0.8075 | 0.6789 - 0.8848 | |
| R_C_alpha_gamma | 0.7224 | 0.5746 - 0.8249 | | 0.7474 | 0.5788 - 0.8488 | | 0.5361 | 0.2206 - 0.7235 | |
| R_C_beta_gamma | 0.7948 | 0.6847 - 0.8708 | | 0.9011 | 0.8349 - 0.9409 | | 0.5456 | 0.2367 - 0.7291 | |
| R_P_delta_theta | 0.9419 | 0.9081 - 0.9641 | | 0.9533 | 0.9188 - 0.9727 | | 0.9205 | 0.8656 - 0.9528 | |
| R_P_delta_alpha | 0.8981 | 0.8269 - 0.9396 | | 0.9731 | 0.9533 - 0.9842 | | 0.8373 | 0.6957 - 0.9089 | |
| R_P_delta_beta | 0.9436 | 0.8901 - 0.9690 | | 0.9534 | 0.9047 - 0.9751 | | 0.9227 | 0.8460 - 0.9581 | |
| R_P_delta_gamma | 0.8288 | 0.7160 - 0.8972 | | 0.7944 | 0.5900 - 0.8888 | | 0.8114 | 0.6852 - 0.8872 | |
| R_P_theta_alpha | 0.8608 | 0.7747 - 0.9152 | | 0.9562 | 0.9268 - 0.9738 | | 0.7884 | 0.6172 - 0.8793 | |
| R_P_theta_beta | 0.926 | 0.8764 - 0.9557 | | 0.9047 | 0.8409 - 0.9430 | | 0.8939 | 0.7859 - 0.9429 | |
| R_P_theta_gamma | 0.7881 | 0.6680 - 0.8682 | | 0.7756 | 0.5913 - 0.8724 | | 0.7482 | 0.5802 - 0.8492 | |
| R_P_alpha_beta | 0.9201 | 0.8772 - 0.9497 | | 0.8669 | 0.7753 - 0.9209 | | 0.9425 | 0.9036 - 0.9656 | |
| R_P_alpha_gamma | 0.7175 | 0.5671 - 0.8217 | | 0.5887 | 0.3082 - 0.7551 | | 0.7337 | 0.5551 - 0.8408 | |
| R_P_beta_gamma | 0.8026 | 0.6954 - 0.8760 | | 0.7502 | 0.5706 - 0.8531 | | 0.7431 | 0.5691 - 0.8467 | |
| R_O_delta_theta | 0.9094 | 0.8611 - 0.9429 | | 0.905 | 0.8384 - 0.9438 | | 0.9005 | 0.8332 - 0.9406 | |
| R_O_delta_alpha | 0.9253 | 0.8832 - 0.9534 | | 0.9575 | 0.9275 - 0.9749 | | 0.8743 | 0.7888 - 0.9251 | |
| R_O_delta_beta | 0.892 | 0.8326 - 0.9324 | | 0.8971 | 0.8098 - 0.9419 | | 0.8125 | 0.6859 - 0.8881 | |
| R_O_delta_gamma | 0.7069 | 0.5513 - 0.8149 | | 0.5688 | 0.2832 - 0.7414 | | 0.6371 | 0.3899 - 0.7837 | |
| R_O_theta_alpha | 0.8876 | 0.8276 - 0.9291 | | 0.9593 | 0.9320 - 0.9756 | | 0.7897 | 0.6495 - 0.8741 | |
| R_O_theta_beta | 0.8607 | 0.7866 - 0.9121 | | 0.8695 | 0.7821 - 0.9219 | | 0.7315 | 0.5498 - 0.8397 | |
| R_O_theta_gamma | 0.6692 | 0.4947 - 0.7909 | | 0.5273 | 0.2198 - 0.7152 | | 0.6028 | 0.3321 - 0.7634 | |
| R_O_alpha_beta | 0.8746 | 0.8076 - 0.9210 | | 0.7984 | 0.6581 - 0.8805 | | 0.8823 | 0.8032 - 0.9297 | |
| R_O_alpha_gamma | 0.7219 | 0.5748 - 0.8244 | | 0.3364 | -0.09276 - 0.5996 | | 0.8885 | 0.8133 - 0.9334 | |
| R_O_beta_gamma | 0.7442 | 0.6087 - 0.8384 | | 0.6196 | 0.3692 - 0.7714 | | 0.7506 | 0.5823 - 0.8511 | |
| R_T_delta_theta | 0.9282 | 0.8866 - 0.9555 | | 0.9302 | 0.8707 - 0.9607 | | 0.893 | 0.8214 - 0.9360 | |
| R_T_delta_alpha | 0.9378 | 0.8973 - 0.9626 | | 0.9687 | 0.9462 - 0.9816 | | 0.8946 | 0.8117 - 0.9394 | |
| R_T_delta_beta | 0.8705 | 0.8013 - 0.9183 | | 0.8886 | 0.8140 - 0.9333 | | 0.7916 | 0.6525 - 0.8752 | |
| R_T_delta_gamma | 0.7081 | 0.5515 - 0.8162 | | 0.8046 | 0.6732 - 0.8832 | | 0.4188 | 0.02617 - 0.6530 | |
| R_T_theta_alpha | 0.9325 | 0.8955 - 0.9577 | | 0.9695 | 0.9489 - 0.9818 | | 0.8761 | 0.7880 - 0.9270 | |
| R_T_theta_beta | 0.9008 | 0.8477 - 0.9375 | | 0.9403 | 0.9003 - 0.9643 | | 0.7993 | 0.6644 - 0.8800 | |
| R_T_theta_gamma | 0.7701 | 0.6480 - 0.8549 | | 0.8729 | 0.7811 - 0.9253 | | 0.5033 | 0.1642 - 0.7042 | |
| R_T_alpha_beta | 0.845 | 0.7625 - 0.9022 | | 0.8302 | 0.7167 - 0.8984 | | 0.7556 | 0.5900 - 0.8542 | |
| R_T_alpha_gamma | 0.7172 | 0.5674 - 0.8214 | | 0.7642 | 0.6013 - 0.8600 | | 0.4498 | 0.07468 - 0.6722 | |
| R_T_beta_gamma | 0.6717 | 0.4971 - 0.7929 | | 0.852 | 0.7505 - 0.9120 | | 0.2671 | -0.2372 - 0.5644 | |

R: relative; F: frontal; C: central; P: parietal; O: occipital; T: temporal; CI: confidence interval

Supplementary Table 2. ICCs of each spectral power feature across each region in dataset 2

|  | Session 1, 2 | 95% CI |
| --- | --- | --- |
| F_Delta | -3.2E-05 | -0.5879 - 0.3702 |
| F_Theta | -0.00002534 | -0.5879 - 0.3702 |
| F_Alpha | -0.00001182 | -0.5879 - 0.3702 |
| F_Beta | -0.00007978 | -0.5880 - 0.3702 |
| F_Gamma | -0.0001011 | -0.5881 - 0.3702 |
| F_All | -0.00004205 | -0.5879 - 0.3702 |
| C_Delta | -0.00003680 | -0.5879 - 0.3702 |
| C_Theta | -0.00003193 | -0.5879 - 0.3702 |
| C_Alpha | -0.00001718 | -0.5879 - 0.3702 |
| C_Beta | -0.0001012 | -0.5880 - 0.3702 |
| C_Gamma | -0.0001271 | -0.5881 - 0.3702 |
| C_All | -0.00005292 | -0.5879 - 0.3702 |
| P_Delta | -0.00003268 | -0.5879 - 0.3702 |
| P_Theta | -0.00003447 | -0.5879 - 0.3702 |
| P_Alpha | -0.00001617 | -0.5879 - 0.3702 |
| P_Beta | -0.0001164 | -0.5881 - 0.3702 |
| P_Gamma | -0.0001509 | -0.5882 - 0.3701 |
| P_All | -5.8E-05 | -0.5880 - 0.3702 |
| O_Delta | -0.00001141 | -0.5879 - 0.3702 |
| O_Theta | -0.000009448 | -0.5879 - 0.3702 |
| O_Alpha | -0.000005436 | -0.5879 - 0.3702 |
| O_Beta | -0.00002738 | -0.5879 - 0.3702 |
| O_Gamma | -0.00003365 | -0.5879 - 0.3702 |
| O_All | -0.00001493 | -0.5879 - 0.3702 |
| T_Delta | -0.00005020 | -0.5880 - 0.3702 |
| T_Theta | -0.00005160 | -0.5879 - 0.3702 |
| T_Alpha | -0.00002555 | -0.5879 - 0.3702 |
| T_Beta | -0.0001753 | -0.5882 - 0.3701 |
| T_Gamma | -0.0002265 | -0.5883 - 0.3701 |
| T_All | -0.00008736 | -0.5880 - 0.3702 |
| F_delta_theta | 0.6114 | 0.3811 - 0.7557 |
| F_delta_alpha | 0.5625 | 0.3032 - 0.7250 |
| F_delta_beta | 0.4184 | 0.07341 - 0.6345 |
| F_delta_gamma | 0.4356 | 0.09990 - 0.6455 |
| F_theta_alpha | 0.8844 | 0.8169 - 0.9271 |
| F_theta_beta | 0.8509 | 0.7622 - 0.9063 |
| F_theta_gamma | 0.8312 | 0.7324 - 0.8936 |
| F_alpha_beta | 0.9008 | 0.8424 - 0.9376 |
| F_alpha_gamma | 0.9103 | 0.8574 - 0.9435 |
| F_beta_gamma | 0.7919 | 0.6691 - 0.8691 |
| C_delta_theta | 0.3831 | 0.01648 - 0.6125 |
| C_delta_alpha | 0.3374 | -0.05620 - 0.5838 |
| C_delta_beta | 0.08487 | -0.4559 - 0.4244 |
| C_delta_gamma | 0.2895 | -0.1329 - 0.5538 |
| C_theta_alpha | 0.9005 | 0.8424 - 0.9373 |
| C_theta_beta | 0.8056 | 0.6922 - 0.8774 |
| C_theta_gamma | 0.8957 | 0.8348 - 0.9342 |
| C_alpha_beta | 0.8606 | 0.7790 - 0.9121 |
| C_alpha_gamma | 0.9083 | 0.8547 - 0.9422 |
| C_beta_gamma | 0.8564 | 0.7722 - 0.9095 |
| P_delta_theta | 0.202 | -0.2749 - 0.4994 |
| P_delta_alpha | 0.2711 | -0.1615 - 0.5420 |
| P_delta_beta | -0.03251 | -0.6431 - 0.3506 |
| P_delta_gamma | 0.2629 | -0.1746 - 0.5368 |
| P_theta_alpha | 0.8901 | 0.8259 - 0.9307 |
| P_theta_beta | 0.8619 | 0.7799 - 0.9132 |
| P_theta_gamma | 0.9028 | 0.8460 - 0.9387 |
| P_alpha_beta | 0.8661 | 0.7872 - 0.9157 |
| P_alpha_gamma | 0.9002 | 0.8406 - 0.9374 |
| P_beta_gamma | 0.8283 | 0.7271 - 0.8919 |
| O_delta_theta | 0.2066 | -0.2679 - 0.5023 |
| O_delta_alpha | 0.3767 | 0.008584 - 0.6079 |
| O_delta_beta | 0.008339 | -0.5797 - 0.3767 |
| O_delta_gamma | 0.3727 | -0.001103 - 0.6062 |
| O_theta_alpha | 0.8449 | 0.7525 - 0.9026 |
| O_theta_beta | 0.912 | 0.8557 - 0.9457 |
| O_theta_gamma | 0.9034 | 0.8465 - 0.9391 |
| O_alpha_beta | 0.8616 | 0.7807 - 0.9127 |
| O_alpha_gamma | 0.8332 | 0.7172 - 0.8991 |
| O_beta_gamma | 0.7941 | 0.6425 - 0.8772 |
| T_delta_theta | 0.04457 | -0.5267 - 0.4007 |
| T_delta_alpha | 0.07829 | -0.4635 - 0.4195 |
| T_delta_beta | -0.05526 | -0.6777 - 0.3359 |
| T_delta_gamma | 0.1033 | -0.4283 - 0.4364 |
| T_theta_alpha | 0.9063 | 0.8492 - 0.9415 |
| T_theta_beta | 0.7818 | 0.6545 - 0.8623 |
| T_theta_gamma | 0.898 | 0.8384 - 0.9357 |
| T_alpha_beta | 0.8606 | 0.7785 - 0.9123 |
| T_alpha_gamma | 0.903 | 0.8460 - 0.9389 |
| T_beta_gamma | 0.7951 | 0.6745 - 0.8710 |
| R_F_Delta | 0.6714 | 0.4778 - 0.7931 |
| R_F_Theta | 0.7106 | 0.5422 - 0.8173 |
| R_F_Alpha | 0.8241 | 0.7203 - 0.8893 |
| R_F_Beta | 0.7114 | 0.5407 - 0.8185 |
| R_F_Gamma | 0.4039 | 0.04984 - 0.6255 |
| R_C_Delta | 0.5478 | 0.2812 - 0.7154 |
| R_C_Theta | 0.8038 | 0.6882 - 0.8765 |
| R_C_Alpha | 0.8014 | 0.6855 - 0.8747 |
| R_C_Beta | 0.748 | 0.6008 - 0.8411 |
| R_C_Gamma | 0.4756 | 0.1660 - 0.6701 |
| R_P_Delta | 0.4154 | 0.07179 - 0.6318 |
| R_P_Theta | 0.8266 | 0.7253 - 0.8906 |
| R_P_Alpha | 0.7244 | 0.5639 - 0.8261 |
| R_P_Beta | 0.7725 | 0.6398 - 0.8564 |
| R_P_Gamma | 0.413 | 0.06395 - 0.6313 |
| R_O_Delta | 0.4736 | 0.1647 - 0.6684 |
| R_O_Theta | 0.8414 | 0.7468 - 0.9004 |
| R_O_Alpha | 0.7535 | 0.6096 - 0.8445 |
| R_O_Beta | 0.7774 | 0.6470 - 0.8597 |
| R_O_Gamma | 0.5768 | 0.3293 - 0.7331 |
| R_T_Delta | 0.4448 | 0.1145 - 0.6513 |
| R_T_Theta | 0.7865 | 0.6610 - 0.8656 |
| R_T_Alpha | 0.7889 | 0.6650 - 0.8670 |
| R_T_Beta | 0.6515 | 0.4483 - 0.7801 |
| R_T_Gamma | 0.4903 | 0.1892 - 0.6794 |
| R_F_delta_theta | 0.6486 | 0.4441 - 0.7781 |
| R_F_delta_alpha | 0.8566 | 0.7724 - 0.9096 |
| R_F_delta_beta | 0.6027 | 0.3681 - 0.7500 |
| R_F_delta_gamma | 0.6573 | 0.4577 - 0.7837 |
| R_F_theta_alpha | 0.7474 | 0.5992 - 0.8409 |
| R_F_theta_beta | 0.3047 | -0.1038 - 0.5621 |
| R_F_theta_gamma | 0.5476 | 0.2823 - 0.7149 |
| R_F_alpha_beta | 0.6362 | 0.4207 - 0.7713 |
| R_F_alpha_gamma | 0.7763 | 0.6444 - 0.8592 |
| R_F_beta_gamma | 0.4057 | 0.05218 - 0.6267 |
| R_C_delta_theta | 0.7187 | 0.5525 - 0.8231 |
| R_C_delta_alpha | 0.8048 | 0.6896 - 0.8771 |
| R_C_delta_beta | 0.4607 | 0.1431 - 0.6605 |
| R_C_delta_gamma | 0.7066 | 0.5335 - 0.8154 |
| R_C_theta_alpha | 0.7551 | 0.6108 - 0.8458 |
| R_C_theta_beta | 0.5812 | 0.3339 - 0.7366 |
| R_C_theta_gamma | 0.4655 | 0.1494 - 0.6638 |
| R_C_alpha_beta | 0.6731 | 0.4830 - 0.7936 |
| R_C_alpha_gamma | 0.6671 | 0.4737 - 0.7898 |
| R_C_beta_gamma | 0.5176 | 0.2328 - 0.6965 |
| R_P_delta_theta | 0.6841 | 0.4975 - 0.8013 |
| R_P_delta_alpha | 0.8141 | 0.7043 - 0.8830 |
| R_P_delta_beta | 0.2911 | -0.1256 - 0.5535 |
| R_P_delta_gamma | 0.6252 | 0.4051 - 0.7639 |
| R_P_theta_alpha | 0.8512 | 0.7637 - 0.9063 |
| R_P_theta_beta | 0.7136 | 0.5454 - 0.8196 |
| R_P_theta_gamma | 0.5209 | 0.2373 - 0.6988 |
| R_P_alpha_beta | 0.7523 | 0.6066 - 0.8441 |
| R_P_alpha_gamma | 0.6477 | 0.4434 - 0.7774 |
| R_P_beta_gamma | 0.5528 | 0.2901 - 0.7184 |
| R_O_delta_theta | 0.5828 | 0.3391 - 0.7368 |
| R_O_delta_alpha | 0.8305 | 0.7307 - 0.8933 |
| R_O_delta_beta | 0.553 | 0.2893 - 0.7187 |
| R_O_delta_gamma | 0.7568 | 0.6134 - 0.8469 |
| R_O_theta_alpha | 0.8662 | 0.7874 - 0.9157 |
| R_O_theta_beta | 0.8615 | 0.7801 - 0.9128 |
| R_O_theta_gamma | 0.7165 | 0.5489 - 0.8217 |
| R_O_alpha_beta | 0.7623 | 0.6234 - 0.8501 |
| R_O_alpha_gamma | 0.6303 | 0.4128 - 0.7672 |
| R_O_beta_gamma | 0.6114 | 0.3864 - 0.7544 |
| R_T_delta_theta | 0.7102 | 0.5397 - 0.8175 |
| R_T_delta_alpha | 0.8424 | 0.7497 - 0.9008 |
| R_T_delta_beta | 0.5833 | 0.3382 - 0.7376 |
| R_T_delta_gamma | 0.6947 | 0.5149 - 0.8079 |
| R_T_theta_alpha | 0.8196 | 0.7133 - 0.8864 |
| R_T_theta_beta | 0.5281 | 0.2490 - 0.7033 |
| R_T_theta_gamma | 0.7655 | 0.6277 - 0.8523 |
| R_T_alpha_beta | 0.63 | 0.4117 - 0.7672 |
| R_T_alpha_gamma | 0.7521 | 0.6066 - 0.8438 |
| R_T_beta_gamma | 0.5367 | 0.2641 - 0.7083 |

R: relative; F: frontal; C: central; P: parietal; O: occipital; T: temporal; CI: confidence interval
